# TREM-1 promoted apoptosis and inhibited autophagy in LPS-treated HK-2 cells through the NF-κB pathway

**DOI:** 10.7150/ijms.50893

**Published:** 2021-01-01

**Authors:** Pan Pan, Xudong Liu, LingLing Wu, Xiaogang Li, Kaifei Wang, Xiaoting Wang, Xiang Zhou, Yun Long, Dawei Liu, Lixin Xie, Longxiang Su

**Affiliations:** 1 College of Pulmonary and Critical Care Medicine, Chinese PLA General Hospital, 17th Heishanhujia, Haidian District, Beijing 100091, China; 2 Department of Critical Care Medicine, Peking Union Medical College Hospital, Peking Union Medical College & Chinese Academy of Medical Sciences, Beijing 100730, China; 3 Medical Science Research Center, Peking Union Medical College Hospital, Peking Union Medical College & Chinese Academy of Medical Sciences, Beijing 100730, China; 4 Department of Nephrology, Chinese PLA General Hospital, Chinese PLA Institute of Nephrology, State Key Laboratory of Kidney Diseases, National Clinical Research Center for Kidney Diseases, Beijing 100853, China

**Keywords:** Sepsis, AKI, TREM-1, Apoptosis, Autophagy, NF-κB signaling pathway

## Abstract

Triggering receptor expressed by myeloid cells (TREM-1) is an amplifier of inflammatory responses triggered by bacterial or fungal infection. Soluble TREM-1 (sTREM-1) expression was found to be upregulated in sepsis-associated acute kidney injury (SA-AKI) and predicted to be a potential biomarker. However, the mechanism remains unclear. The human kidney-2 (HK-2) cell line was treated with lipopolysaccharide (LPS) and used to examine the potential roles of TREM-1 in apoptosis and autophagy. A cell viability assay was employed to assess the number of viable cells and as a measure of the proliferative index. The concentrations of sTREM-1, interleukin (IL)-1β, tumor necrosis factor-α (TNFα) and IL-6 in cell-free culture supernatants were measured by enzyme-linked immunosorbent assay (ELISA). Western blot analysis was performed to analyze apoptosis, autophagy and the relevant signaling pathways. The results suggested that TREM-1 overexpression after LPS treatment decreased proliferation and increased apoptosis. The concentrations of sTREM-1, IL-1β, TNFα and IL-6 in cell-free culture supernatants were increased in the TREM-1 overexpression group after LPS treatment. Expression of the antiapoptotic gene Bcl-2 was downregulated in the TREM-1 overexpression group, while that of the proapoptotic genes Bax, cleaved caspase-3 and cleaved caspase-9 was upregulated. Overexpression of TREM-1 downregulated expression of the autophagy genes Beclin-1, Atg-5 and LC3b and increased the gene expression of p62, which inhibits autophagy. Conversely, treatment with TREM-1-specific shRNA had the opposite effects. The nuclear factor-κB (NF-κB) signaling pathway (P-p65/p65 and P-IκBα/IκBα) in LPS-induced HK-2 cells was regulated by TREM-1. In summary, TREM-1 promoted apoptosis and inhibited autophagy in HK-2 cells in the context of LPS exposure potentially through the NF-κB pathway.

## Introduction

Sepsis is the leading cause of death in patients admitted to the intensive care unit, and death from sepsis is caused by a deleterious immune response to infection 1. Severe sepsis is associated with multiorgan dysfunction, but the mechanisms leading to this dysfunction remain unclear 2. The kidney is the organ most commonly affected by sepsis, leading to sepsis-associated acute kidney injury (SA-AKI). Acute kidney injury (AKI) develops in up to 60% of patients with sepsis, and up to 50% of patients with AKI have sepsis 3. Earlier recognition, diagnosis and treatment can improve renal outcomes and decrease mortality 4. According to recent studies, soluble triggering receptor expressed on myeloid cell-1 (sTREM-1) may be a relatively sensitive and accurate biomarker for the diagnosis of sepsis and infectious diseases 5, 6. We found that urine sTREM-1 could be used to diagnose sepsis or even provide an early warning of possible secondary AKI in sepsis patients 7, 8.

sTREM-1 is a 17-kDa fragment cleaved from triggering receptor expressed on myeloid cell-1 (TREM-1) by a metalloproteinase 9. The TREM family is a recently discovered family whose members are expressed on the cell surface and play important roles in innate and adaptive immunity 10. Among the six identified TREM proteins (TREM-1, TREM-2, and TREML1-4), TREM-1 is a transmembrane glycoprotein expressed on monocytes, neutrophils and macrophages 11-13. TREM-1 is an amplifier of inflammatory and immune responses that functions by mediating cross-talk with TLRs and/or NLRs, which are predominantly associated with bacterial and fungal infection 14. TREM-1 expression was found to be significantly increased in response to lipopolysaccharide (LPS), subsequently promoting the release of various proinflammatory cytokines, such as interleukins (ILs; including IL-1β) and tumor necrosis factor-α (TNF-α) 12. Knockdown of TREM-1 expression inhibited the activity of the nuclear factor-κB (NF-κB) pathway 15. Sepsis induced the activation of granulocytes and monocytes/macrophages, which highly express TREM-1.

The inflammatory response during AKI damages tubular epithelial cells (TECs) 16, with significant damage and necrosis observed in the proximal tubule. The proinflammatory response mediated by TEC activation was shown to activate dendritic cells, produce chemokines, and activate T lymphocyte cells 17. In addition, activated TECs directly interact with neutrophils, monocytes and T cells 18. TREM-1 expression was also reported to be increased in TECs after ischemia-reperfusion injury (IRI). TEC-associated TREM-1 in IRI was shown to impact cell fate and metabolism 19. A previous study reported that sTREM-1 levels were significantly increased at the time of diagnosis and 24 h before AKI diagnosis 8, 20. We hypothesized that the local inflammatory response in the kidney contributes to the elevated secretion of urine sTREM-1 during SA-AKI 7. However, the function of TREM-1 in SA-AKI remains unclear. In the present work, we examined the potential roles of TREM-1 in apoptosis and autophagy in the human kidney-2 (HK-2) cell line treated with LPS to determine how TREM-1 damages parenchymal cells in the kidneys.

## Materials and Methods

### Cell culture and treatment

HK-2 human renal proximal TECs (American Type Culture Collection, Manassas, VA, USA) were cultured in Dulbecco's modified Eagle's medium and Ham's F-12 medium (DMEM/F12, Gibco) supplemented with 10% fetal bovine serum, 100 U/mL penicillin, and 100 mg/mL streptomycin. HK-2 cells at logarithmic growth phase were digested with trypsin (Solarbio, T1300-100), seeded in a 6-well plate (3×10^5^/well), and then cultured for another 8 h before treatment.

For TREM-1 depletion studies, TREM-1-specific siRNA (Santa Cruz, Cat# sc-42999), control (CON) shRNA plasmid (Santa Cruz, Cat# sc-108060), TREM-1 cDNA ORF clone (Sino Bio, Cat# HG10511-UT), or negative control vector (Sino Bio, Cat# CV011) was transfected into HK-2 cells using Lipofectamine 3000 (Thermo Fisher). Cells were grown to a subconfluent density at 37°C with 5% CO_2_ and stimulated with LPS (100 ng/mL).

### Cell viability assay

The Cell Counting Kit-8 (CCK8) assay was employed to assess the number of viable cells and as a measure of the proliferation index. HK-2 cells were plated in 96-well plates at a density of 1×10^5^ cells/well and then cultured in complete medium with or without LPS (100 ng/ml). The optical density at 450 nm (OD450) was determined at each time point.

### qRT-PCR

Total mRNA was extracted from primary monocytes using the RNeasy® Micro Kit (QIAGEN). RNA quantity was measured with a Nanodrop™ 2000C spectrophotometer (Thermo Fisher Scientific) and reverse transcribed using iScript™ Reverse Transcription SuperMix for qRT-PCR (Bio-Rad) according to the manufacturer's instructions. The RT-PCR conditions used for all reactions were as follows: 25°C, 5 min/46°C, 20 min/95°C, and 1 min per cycle. Reverse transcription was performed on a MyiQ thermocycler (Bio-Rad). The relative expression of TREM-1 in HK-2 cells was then analyzed by qPCR using Sso advanced Universal SYBR Green Supermix (Bio-Rad) and human TREM-1 probes (QuantiTect Primers Assay, QIAGEN). GAPDH served as a housekeeping control gene. qPCR was performed on a MyiQ thermocycler, and the results were quantified with iQ5 software (QIAGEN). The expression levels of each target gene in each sample were calculated by the comparative Ct method after normalization to the expression levels of the housekeeping gene.

### Enzyme-linked immunosorbent assay (ELISA)

The concentrations of sTREM-1, IL-1β, TNFα and IL-6 in cell-free culture supernatants were measured using commercially available specific ELISA kits (DuoSet, R&D Systems) according to the manufacturer's instructions. The detection limits were 31.3 pg/ml (sTREM-1 and IL-6), 15.6 pg/ml (TNFα) and 3.91 pg/ml (IL-1β).

### Western blot analysis

Western blot analysis was performed using standard procedures. The primary antibodies used were anti-TREM-1, anti-caspase 3 and anti-caspase 9 antibodies (Abcam, Cambridge, MA, USA) and anti-ATG5, anti-BECN1, anti-LC3 and anti-P62 (Santa Cruz Biotechnology, Santa Cruz, CA, USA). Horseradish peroxidase-conjugated secondary antibodies were used, and specific antibody-antigen complexes were detected using a chemiluminescent substrate.

### Statistical analyses

Experiments were performed at least in triplicate, and the data are expressed as the mean ± standard deviation (SD). For statistical analyses, the data were analyzed using Student's t-test. *P < 0.05* was used to indicate significance. One-way ANOVA was used for comparisons among different groups.

## Results

### TREM-1 expression in HK-2 cells was induced following LPS stimulation

**Figure 1:**
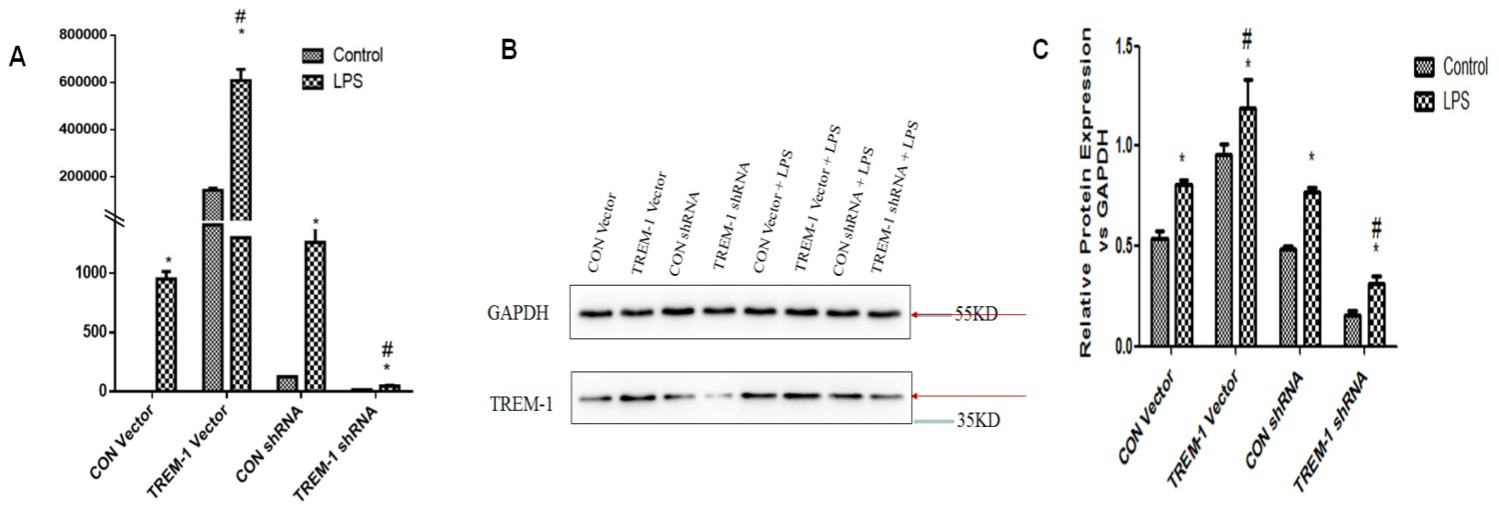
TREM-1 expression in HK-2 cells treated with a TREM-1 vector or shRNA with or without LPS. HK-2 cells were transfected with a control vector, plasmid for TREM-1 expression, control siRNA or TREM-1-specific siRNA as described in the Materials and Methods section. Panel A: Representative PT-PCR results indicating suppression of TREM-1 by siRNA (TREM-1 siRNA) or overexpression of TREM-1 (TREM-1 DNA). Panels B & C: Representative immunoblotting results indicating suppression of TREM-1 by siRNA (TREM-1 siRNA) or overexpression of TREM-1 (TREM-1 DNA) from qualitative or quantitative analysis, respectively. * *P < 0.05* compared with control cells without LPS; # *P < 0.05* compared with control cells with LPS. The data presented are one representative assessment of triplicate assays with similar results.

RT-PCR and western blotting were performed to evaluate TREM-1 gene and protein expression, respectively, in HK-2 cells after TREM-1 vector or shRNA transfection (Figure 1). Both the gene and protein levels (protein ratio in the treated vs. control group) revealed that TREM-1 expression was significantly increased in cells transfected with the TREM-1 vector but decreased in cells transfected with TREM-1-shRNA (CON vs. TREM-1 vector vs. TREM-1-shRNA, *P < 0.05*). These results demonstrated the successful overexpression or suppression of TREM-1 in HK-2 cells. As shown in the same figure, LPS stimulated TREM-1 expression in HK-2 cells, as evidenced by increased gene and protein expression in the LPS-treated group compared to the vector and shRNA control groups (LPS gene + CON vector vs. LPS + CON shRNA vs. vector vs. shRNA group, 950.40±64.01 vs. 1260.07±129.91 vs. 143656.67±9916.67 vs. 126.28±14.96 vs. 1±0; LPS protein vs. vector vs. shRNA group vs. control, 0.81±0.02 vs. 0.77±0.03, 0.54±0.03 vs. 0.49±0.02 vs. 0.5±0.03, both *P < 0.05*). TREM-1 expression was further augmented by LPS in cells transfected with the TREM-1 vector (LPS gene + CON vector group vs. LPS + TREM-1 vector group, 950.40±64.01 vs. 608376.67±47177.31; LPS protein vs. LPS + TREM-1 vector group 0.96±0.05 vs. 1.19±0.15, *P < 0.05*) but attenuated in cells transfected with TREM-1-specific shRNA even in the presence of LPS (LPS gene + CON shRNA group vs. LPS + TREM-1 shRNA group, 1260.07±129.91 vs. 45.35±7.37; LPS protein vs. LPS + TREM-1 shRNA group, 0.77±0.03 vs. 0.32±0.03, *P < 0.05*).

### Cell viability and apoptosis in response to LPS and TREM-1 intervention

**Figure 2:**
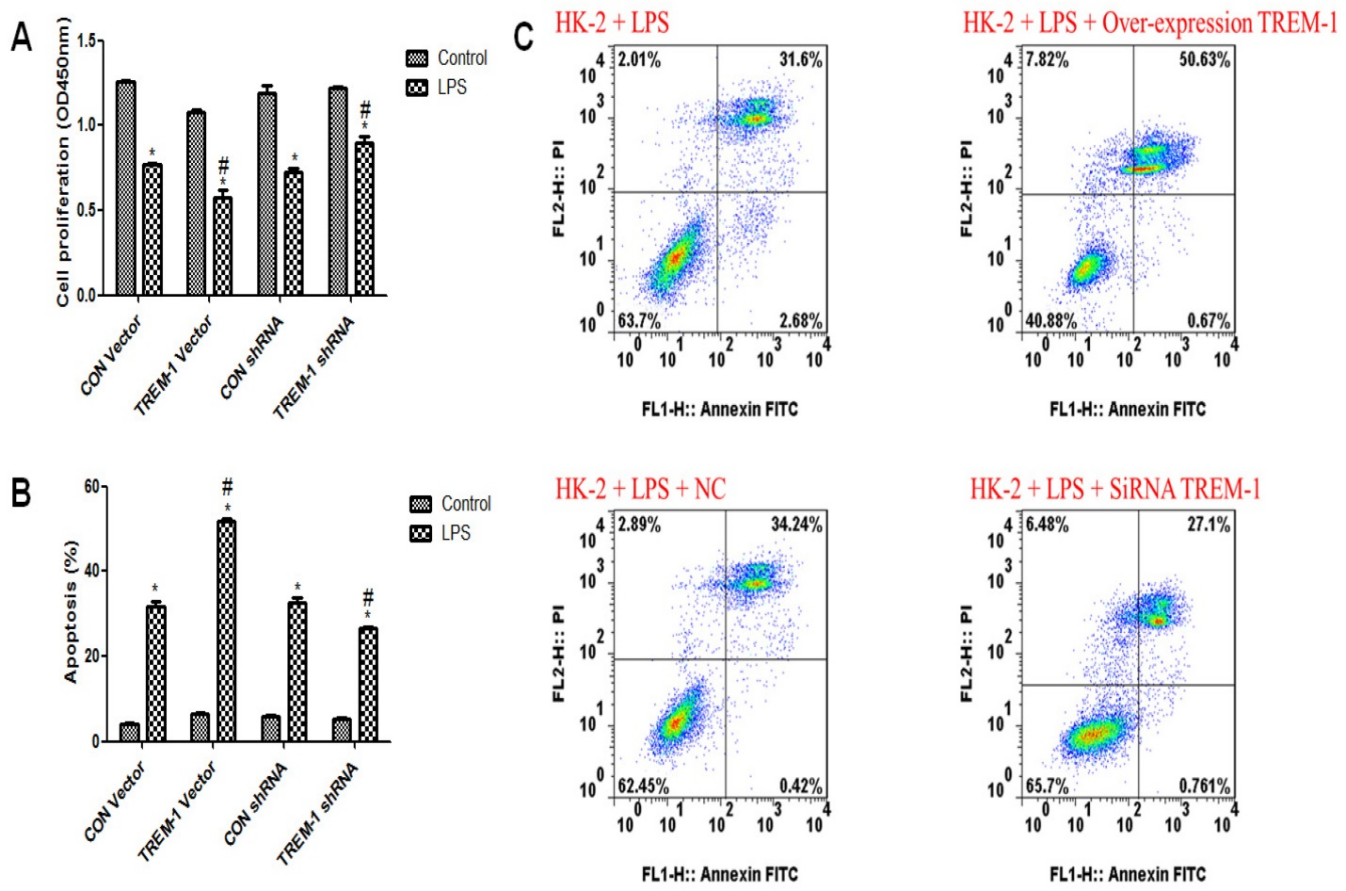
The roles of TREM-1 in modulating HK-2 cell proliferation and apoptosis in response to LPS. HK-2 cells were transfected with a control vector, TREM-1 vector, control shRNA, or TREM-1-specific shRNA as described in the Materials and Methods section. The cells were then cultured for 72 h with or without LPS (10 µg/mL). Panel A: Quantitative comparison of cell proliferation in cells with or without LPS exposure. Panel B: Quantitative comparison of cell apoptosis in cells with or without LPS exposure. Panel C: Flow cytometry of cell apoptosis in cells with or without LPS exposure. * *P < 0.05* compared with control cells without LPS; # *P < 0.05* compared with control cells with LPS. The data presented are a representative assessment of triplicate assays with similar results.

As shown in Figure 2A, HK-2 cell viability was decreased in response to LPS treatment (CON vector vs. CON vector+LPS, 1.26±0.02 vs. 0.77±0.02; CON shRNA vs. CON shRNA+LPS, 1.19±0.08 vs. 0.72±0.04, all *P<0.05*). Figure 2B & C illustrates increased apoptosis in response to LPS treatment (CON vector vs. CON vector+LPS, 4.24±0.28 vs. 31.71±2.11; CON shRNA vs. CON shRNA+LPS, 5.93±0.25 vs. 32.59±2.05, all *P<0.05*). After TREM-1 vector or shRNA treatment, the TREM-1 shRNA+LPS group exhibited increased cell proliferation compared to the CON shRNA+LPS group (0.90±0.06 vs. 0.72±0.04, *P<0.05*), while the TREM-1 vector+LPS group exhibited decreased cell proliferation compared to the CON vector+LPS group (0.58±0.08 vs. 0.77±0.02, *P<0.05*). However, the ratio of apoptotic cells showed the opposite trend: the apoptosis rate in the TREM-1 shRNA+LPS group was lower than that in the CON shRNA+LPS group (26.64±0.60 vs. 32.59±2.05, *P<0.05*), while that in the TREM-1 vector+LPS group was higher than that in the CON vector+LPS group (51.79±1.08 vs. 31.71±2.11, *P<0.05*).

### Cytokine expression in HK-2 cells was regulated by TREM-1 after LPS treatment

**Figure 3:**
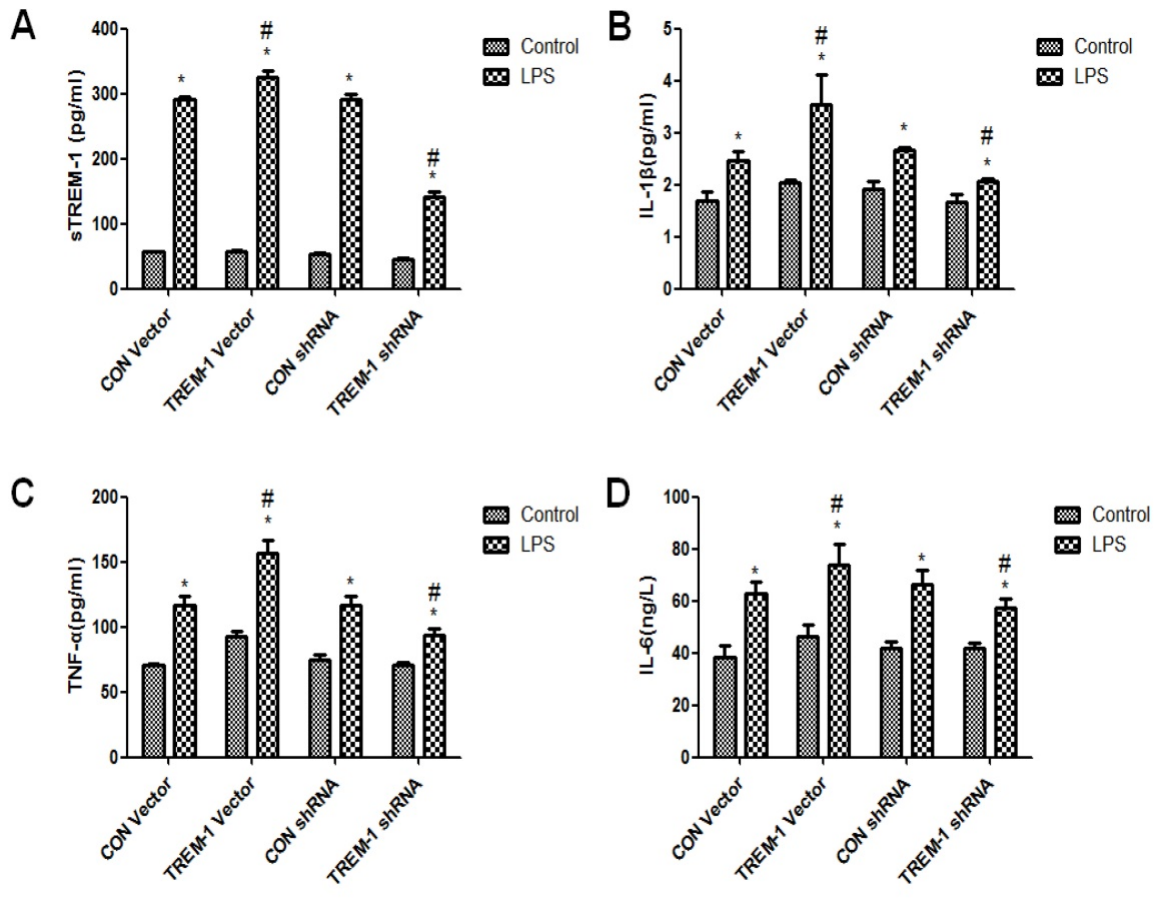
Inflammation and cytokine levels in supernatants of HK-2 cells treated with a TREM-1 vector or shRNA with or without LPS. The concentrations of sTREM-1 (panel A), IL-1β (panel B), TNFα (panel C), and IL-6 (panel D) were measured. HK-2 cells were transfected with a control vector, plasmid for TREM-1 expression, control siRNA or TREM-1-specific siRNA as described in the Materials and Methods section. * *P < 0.05* compared with control cells without LPS; # *P < 0.05* compared with control cells with LPS. The data presented are a representative assessment of triplicate assays with similar results.

The cross-linking of TREM-1 was shown to stimulate cytokine secretion in monocytes and neutrophils. Thus, we investigated whether TREM-1 expression in HK-2 cells is related to the production of IL-1β, TNFα and IL-6 after LPS treatment (Figure 3). As shown in Figure 3, sTREM-1 levels were increased after LPS treatment in all groups (CON vector vs. CON vector+LPS, 56.85±0.68 vs. 292.13±3.40; CON shRNA vs. CON shRNA+LPS, 53.12±1.97 vs. 292.13±6.80; TREM-1 vector vs. TREM-1 vector+LPS, 57.38±1.06 vs. 326.06±9.00; TREM-1 shRNA vs. TREM-1 shRNA+LPS, 44.97±1.98; all *P<0.05*). However, the TREM-1 vector+LPS group had a significantly higher sTREM-1 level than the CON vector+LPS group, while the TREM-1 shRNA+LPS group had a significantly lower sTREM-1 level than the CON shRNA+LPS group. The expression levels of other cytokines showed the same trend; IL-1β (CON vector vs. CON vector+LPS, 1.69±0.17 vs. 2.47±0.17; CON shRNA vs. CON shRNA+LPS, 1.91±0.15 vs. 2.66±0.07; TREM-1 vector vs. TREM-1 vector+LPS, 2.04±0.06 vs. 3.54±0.58; TREM-1 shRNA vs. TREM-1 shRNA+LPS, 1.66±0.15 vs. 2.07±0.04; all *P<0.05*), TNF-α (CON vector vs. CON vector+LPS, 70.87±1.25 vs. 92.40±4.02; CON shRNA vs. CON shRNA+LPS, 74.77±3.94 vs. 117.08±6.76; TREM-1 vector vs. TREM-1 vector+LPS, 92.40±4.02 vs. 156.43±10.07; TREM-1 shRNA vs. TREM-1 shRNA+LPS, 70.91±2.20 vs. 93.90±4.50; all *P<0.05*), and IL-6 levels (CON vector vs. CON vector+LPS, 38.10±4.75 vs. 62.65±4.50; CON shRNA vs. CON shRNA+LPS, 41.68±2.52 vs. 66.31±5.69; TREM-1 vector vs. TREM-1 vector+LPS, 46.38±4.25 vs. 73.97±7.71; TREM-1 shRNA vs. TREM-1 shRNA+LPS, 41.75±2.21 vs. 57.23±3.81; all *P<0.05*) were increased in the LPS-treated group compared with the control group without LPS. In addition, IL-1β, TNF-α, and IL-6 levels were significantly increased after TREM-1 vector transfection and significantly decreased after TREM-1-specific shRNA transfection.

### LPS-induced HK-2 cell apoptosis was mediated by TREM-1

**Figure 4:**
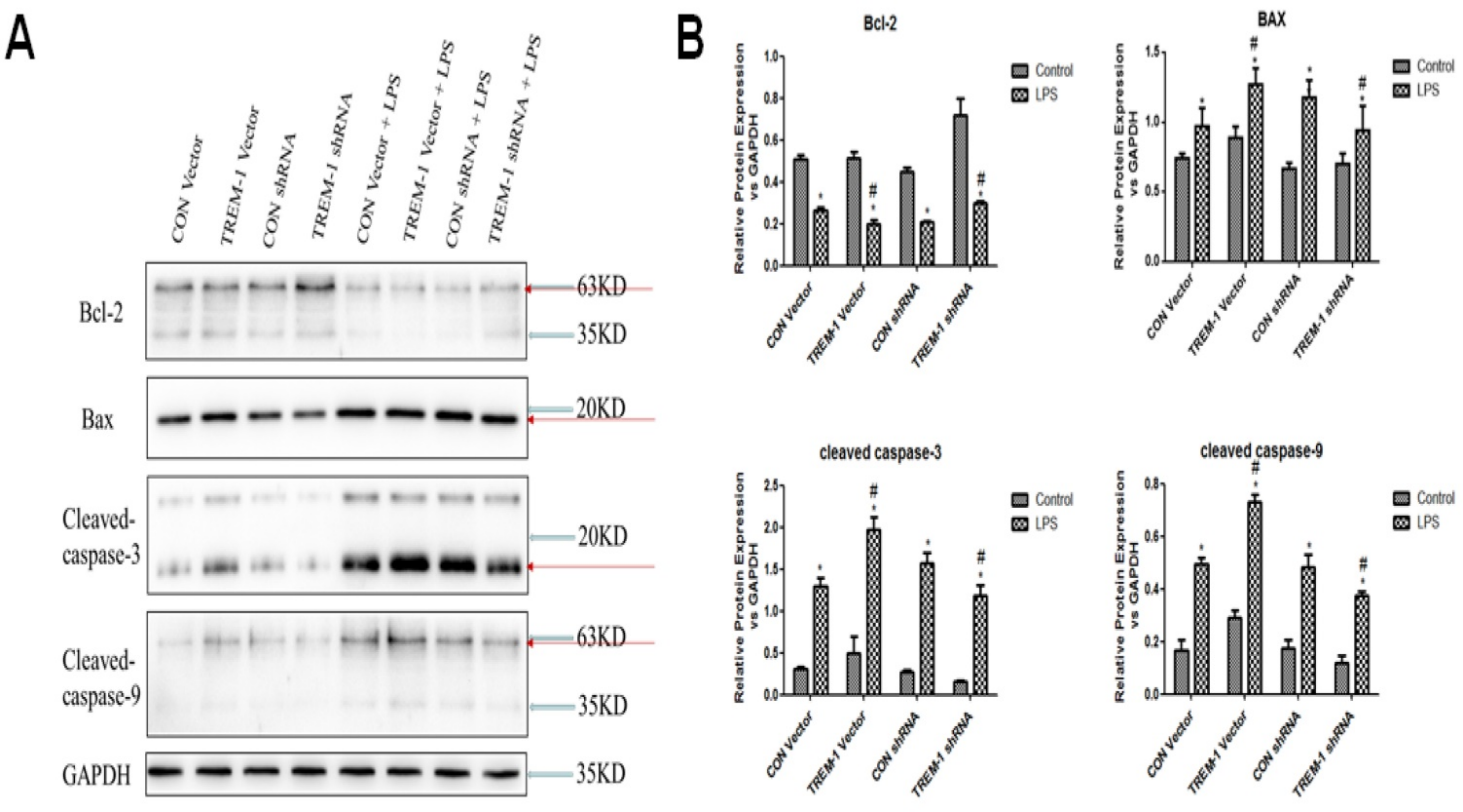
LPS-induced HK-2 cell apoptosis was mediated by TREM-1. HK-2 cells were transfected with a plasmid containing a negative control vector/TREM-1 gene or negative control shRNA/TREM-1-specific shRNA, followed by exposure to LPS (10 µg/mL). Bcl-2, Bax, cleaved caspase-3, and cleaved caspase-9 were assessed by immunoblotting as described in the Materials and Methods section. Panel A: Representative images showing the results of immunoblotting. Panel B: Average of three separate immunoblots for Bcl-2, Bax, cleaved caspase-3, and cleaved caspase-9. Vertical axis: ratio to control; horizontal axis: cells transfected with plasmids containing negative control vector/TREM-1 gene or negative control shRNA/TREM-1 shRNA. * *P< 0.05*.

Regarding the expression of apoptosis-associated proteins, as shown in Figure 4, Bcl-2 expression was decreased in cells stimulated with LPS in all groups (CON vector vs. CON vector+LPS, 0.51±0.02 vs. 0.26±0.01; CON shRNA vs. CON shRNA+LPS, 0.45±0.01 vs. 0.21±0.01; TREM-1 vector vs. TREM-1 vector+LPS, 0.51±0.03 vs. 0.20±0.02; TREM-1 shRNA vs. TREM-1 shRNA+LPS, 0.72±0.08 vs. 0.30±0.01; all *P<0.05*). However, the TREM-1 vector+LPS group had a significantly lower Bcl-2 level than the CON vector+LPS group, while the TREM-1 shRNA+LPS group had a significantly higher Bcl-2 level than the CON shRNA+LPS group. In contrast, the expression patterns of Bax, cleaved caspase-3 and cleaved caspase-9 differed. After LPS treatment, Bax (CON vector vs. CON vector+LPS, 0.74±0.04 vs. 0.97±0.13; CON shRNA vs. CON shRNA+LPS, 0.67±0.04 vs. 1.18±0.12; TREM-1 vector vs. TREM-1 vector+LPS, 0.89±0.08 vs. 1.27±0.12; TREM-1 shRNA vs. TREM-1 shRNA+LPS, 0.70±0.07 vs. 0.94±0.18; all *P<0.05*), cleaved caspase-3 (CON vector vs. CON vector+LPS, 0.31±0.03 vs. 1.30±0.10; CON shRNA vs. CON shRNA+LPS, 0.28±0.0.02 vs. 1.57±0.12; TREM-1 vector vs. TREM-1 vector+LPS, 0.50±0.19 vs. 1.98±0.15; TREM-1 shRNA vs. TREM-1 shRNA+LPS, 0.16±0.01 vs. 1.19±0.12; all *P<0.05*) and cleaved caspase-9 (CON vector vs. CON vector+LPS, 0.17±0.04 vs. 0.49±0.02; CON shRNA vs. CON shRNA+LPS, 0.17±0.03 vs. 0.49±0.05; TREM-1 vector vs. TREM-1 vector+LPS, 0.29±0.03 vs. 0.73±0.03; TREM-1 shRNA vs. TREM-1 shRNA+LPS, 0.12±0.03 vs. 0.38±0.02; all *P<0.05*) levels were significantly increased. However, the TREM-1 vector+LPS group had significantly higher Bax, cleaved caspase-3 and cleaved caspase-9 levels than the CON vector+LPS group, while the TREM-1 shRNA+LPS group had significantly lower Bax, cleaved caspase-3 and cleaved caspase-9 levels than the CON shRNA+LPS group.

### LPS-induced HK-2 cell autophagy was mediated by TREM-1

**Figure 5:**
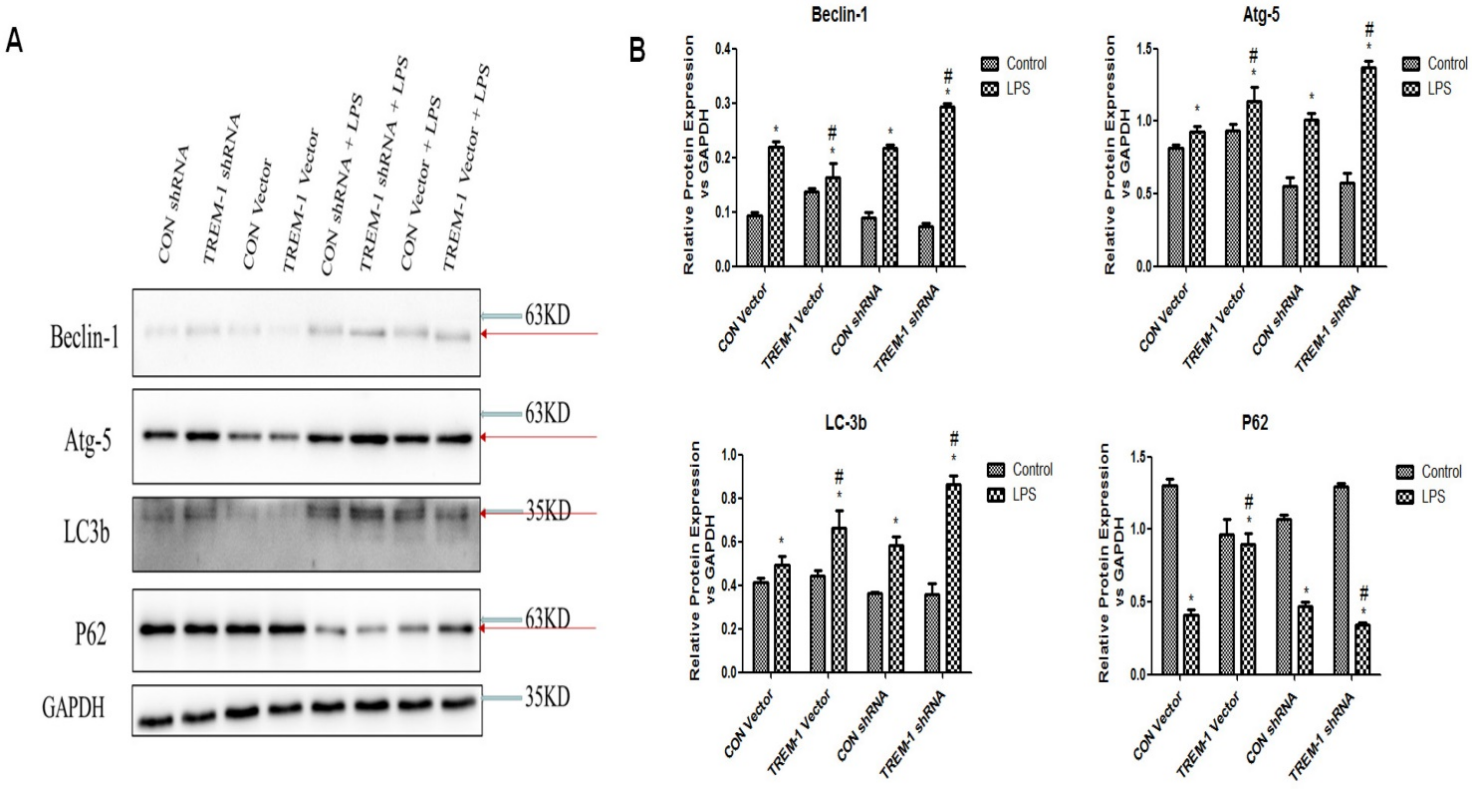
LPS-induced HK-2 cell autophagy was mediated by TREM-1. HK-2 cells were transfected with a plasmid containing a negative control vector/TREM-1 gene or negative control shRNA/TREM-1-specific shRNA, followed by exposure to LPS (10 µg/mL). Beclin-1, Atg-5, LC-3b, and p62 were assessed by immunoblotting as described in the Materials and Methods section. Panel A: Representative images of immunoblots. Panel B: Averages of three separate immunoblots for Beclin-1, Atg-5, LC-3b, and p62. Vertical axis: ratio to control; horizontal axis: cells transfected with plasmids containing a negative control vector/TREM-1 gene or negative control shRNA/TREM-1-specific shRNA. * *P< 0.05*.

Autophagy was further assessed by western blot analysis of expression of the autophagy-related genes Beclin1, ATG5, LC3B-II and p62 in cells treated with or without LPS, as shown in Figure 5. The expression of Beclin1, ATG5, and LC3B-II was increased in the cells stimulated with LPS in all groups (Beclin: CON vector vs. CON vector+LPS, 0.10±0.01 vs. 0.22±0.01; CON shRNA vs. CON shRNA+LPS, 0.09±0.01 vs. 0.22±0.01; TREM-1 vector vs. TREM-1 vector+LPS, 0.14±0.01 vs. 0.16±0.02; TREM-1 shRNA vs. TREM-1 shRNA+LPS, 0.07±0.01 vs. 0.29±0.01; all *P<0.05*; Atg 5: CON vector vs. CON vector+LPS, 0.81±0.02 vs. 0.93±0.04; CON shRNA vs. CON shRNA+LPS, 0.55±0.06 vs. 1.01±0.05; TREM-1 vector vs. TREM-1 vector+LPS, 0.94±0.05 vs. 1.14±0.09; TREM-1 shRNA vs. TREM-1 shRNA+LPS, 0.57±0.07 vs. 1.37±0.05; all *P<0.05*; LC3B: CON vector vs. CON vector+LPS, 0.42±0.02 vs. 0.49±0.04; CON shRNA vs. CON shRNA+LPS, 0.16±0.01 vs. 0.58±0.04; TREM-1 vector vs. TREM-1 vector+LPS, 0.44±0.02 vs. 0.66±0.08; TREM-1 shRNA vs. TREM-1 shRNA+LPS, 0.19±0.01 vs. 0.86±0.04; all *P<0.05*). However, p62 expression followed the opposite trend (CON vector vs. CON vector+LPS, 1.30±0.05 vs. 0.41±0.05; CON shRNA vs. CON shRNA+LPS, 1.07±0.03 vs. 0.47±0.03; TREM-1 vector vs. TREM-1 vector+LPS, 0.36±0.05 vs. 0.90±0.07; TREM-1 shRNA vs. TREM-1 shRNA+LPS, 1.30±0.02 vs. 0.34±0.01; all *P<0.05*). Densitometric analysis of the protein bands revealed that as expected, overexpression of TREM-1 in the LPS-treated group induced the expression of autophagy-related genes and had the opposite effect on the degradation-related protein p62. In LPS-treated cells, the expression of autophagy-related genes was further upregulated, and p62 expression was further downregulated compared with that in control cells.

### The transcriptional level of NF-κB was regulated by TREM-1 in HK-2 cells

**Figure 6:**
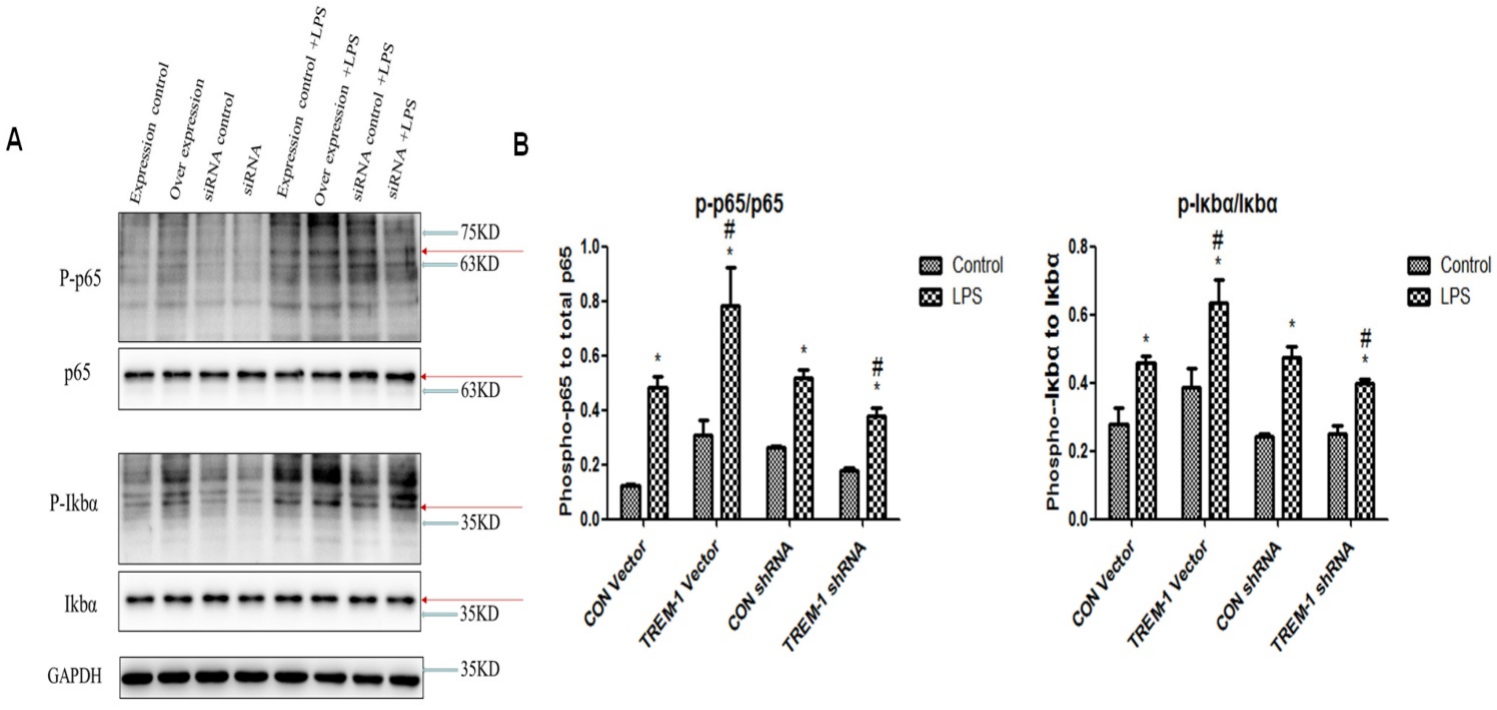
The NF-κB signaling pathway in LPS-treated HK-2 cells was regulated by TREM-1. HK-2 cells were transfected with plasmid containing a negative control vector/TREM-1 gene or negative control shRNA/TREM-1-specific shRNA, followed by exposure to LPS (10 µg/mL). P-p65, p65, P-IκBα, and IκBα were assessed by immunoblotting as described in the Materials and Methods section. Panel A: Representative images of immunoblots. Panel B: Average of three separate immunoblots for P-p65/p65 and P-IκBα/IκBα. Vertical axis: ratio to control; horizontal axis: cells transfected with plasmids containing negative control vector/TREM-1 gene or negative control shRNA/TREM-1 shRNA. * *P< 0.05*.

We investigated the role of transcription factors in the induction of TREM-1 expression (Figure 6). Similar to the results for the apoptosis- and autophagy-related proteins, the expression of P-p65, p65, P-IκBα, and IκBα in all the groups was increased after LPS treatment (P-p65/p65: CON vector vs. CON vector+LPS, 0.12±0.01 vs. 0.48±0.05; CON shRNA vs. CON shRNA+LPS, 0.26±0.01 vs. 0.52±0.03; TREM-1 vector vs. TREM-1 vector+LPS, 0.31±0.05 vs. 0.78±0.14; TREM-1 shRNA vs. TREM-1 shRNA+LPS, 0.18±0.01 vs. 0.38±0.03; all *P<0.05*; P-IκBα,/IκBα: CON vector vs. CON vector+LPS, 0.28±0.05 vs. 0.946±0.02; CON shRNA vs. CON shRNA+LPS, 0.24±0.01 vs. 0.48±0.03; TREM-1 vector vs. TREM-1 vector+LPS, 0.38±0.06 vs. 0.63±0.07; TREM-1 shRNA vs. TREM-1 shRNA+LPS, 0.25±0.02 vs. 0.40±0.01; all *P<0.05*). The TREM-1 vector+LPS group had higher P-p65, p65, P-IκBα, and IκBα levels than the CON vector+LPS group. However, the TREM-1 shRNA+LPS group had lower P-p65, p65, P-IκBα, and IκBα levels than the CON shRNA+LPS group.

## Discussion

In the present work, we examined the potential roles of TREM-1 in apoptosis and autophagy in HK-2 cells treated with LPS. Notably, expression of the antiapoptotic gene Bcl-2 was downregulated in the TREM-1 overexpression group, while that of the proapoptotic genes Bax, cleaved caspase-3 and cleaved caspase-9 was upregulated. Overexpression of TREM-1 downregulated expression of the autophagy-related genes Beclin-1, Atg-5 and LC3b and upregulated the gene expression of p62, which inhibits autophagy. Our data further suggested that the changes in apoptosis and autophagy induced by LPS were associated with TREM1-NF-κB inflammation-dependent signaling.

TREM-1 could not only mediate sepsis-related kidney damage but also contribute to inflammation in IgA nephropathy, which has been detected by urinalysis 21. sTREM-1 levels were also shown to be significantly increased in hemodialysis patients and positively correlated with CRP and TNF-α levels 22. In our study, we found that the levels of IL-1β, TNFα and IL-6 were increased after TREM-1 overexpression but decreased after TREM-1 downregulation. A cell proliferation assay showed enhanced cell proliferation after TREM-1-specific siRNA intervention. Our findings are consistent with in vitro studies that demonstrated that TREM-1 blockade by the inhibitory peptide LP17 prolonged the survival of rats that underwent cecum ligation and puncture (CLP) and attenuated systematic inflammatory responses 23. However, some studies have reached the opposite conclusion, instead showing that TREM-1 can attenuate the inflammatory response and reduce tissue damage 24, 25. The mechanism behind this discrepancy remains to be explained.

Our study showed that cell apoptosis was increased after LPS intervention regardless of TREM-1 overexpression or downregulation. From our results, although the level of apoptosis after TREM-1 inhibition was not as high as that after TREM-1 activation, there was no significant difference in the degree of apoptosis. Theoretically, TREM-1 overexpression enhances the increase in mitochondrial ROS caused by LPS, leading to mitochondrial damage, excessive inflammation and apoptosis, while TREM-1 downregulation has the opposite result. In some studies, TREM-1-knockout macrophages exhibited increased apoptosis in response to LPS 26. TREM-1 was also shown to protect monocytes from apoptosis through the regulation of myeloid cell leukemia-1 27. These results showed that different cell types, concentrations of LPS for intervention and durations may influence the role of TREM-1 in apoptosis. Most importantly, autophagy may participate in this biological process. After TREM-1 was inhibited, the level of autophagy in cells was further increased. This result may have been because the regulation of autophagy caused further damage to cells through apoptosis. TREM-1 was reported to modulate autophagic activity and endoplasmic reticulum (ER) stress in colitis in mice 28. Furthermore, TREM-1 in human monocytes/macrophages and epithelial cells was shown in some studies to be regulated by 1α,25-dihydroxyvitamin D3 via mTOR signaling 29. Therefore, we have inferred that TREM-1 may participate in the autophagic process. TREM-1 deficiency could attenuate disease severity and organ injury without affecting pathogen clearance 30.

The NF-κB pathway is an important inflammatory signaling pathway. A number of previous studies have suggested that TREM-1 plays a key role upstream of this signal. TREM-1 was found to play a critical role in osteoarthritis development through the regulation of NF-κB signaling 15. The inhibition of sEH by 1-trifluoromethoxyphenyl-3-(1-propionylpiperidin-4-yl) urea (TPPU) suppressed LPS-induced TREM-1 expression and inflammation by inhibiting NF-κB activation in murine macrophages 31. Wang et al 32 suggested that aggravation of ventilation-induced lung injury (VILI) by TREM-1 in mice was associated with TLR4-MyD88-NF-κB-dependent signaling. Zi et al 33 demonstrated that high sTREM-1 levels were independent predictors of increased endothelial microparticle levels on admission and associated with increased risks for mortality and major adverse cardiovascular events in acute myocardial infarction patients. It was further reported that Sirt6-induced autophagy restricted TREM-1-mediated pyroptosis in ox-LDL-treated endothelial cells. Additionally, TREM-1 and NLRP3 could regulate the inflammatory response of lung macrophages and even improve lung injury 34. TREM-1 was found to aggravate inflammation in acute lung injury (ALI) by activating the NLRP3 inflammasome 35. We found that TREM-1 could regulate NF-κB signaling to influence the processes of apoptosis and autophagy. However, we did not attempt to block NF-κB to evaluate its effect on autophagy. The mechanisms by which NF-κB regulates autophagy and those by which TREM-1 mediates pyroptosis and autophagy remain to be further studied.

## Conclusion

TREM-1 increased the apoptosis of renal TECs and reduced autophagy by mediating LPS intervention. Imbalance between apoptosis and autophagy is one of the core mechanisms of kidney injury after sepsis. Blockade of TREM-1 resulted in decreased apoptosis and increased autophagy, which may have been due to an increase in inflammatory damage caused by modulation of the NF-κB pathway. The TREM-1 receptor may indirectly affect mitochondria through its interactions with ligands, thereby enhancing inflammation, or directly affect mitochondria through signal transduction pathways. TREM-1 may be a key target for intervention in renal function damage, but further work is needed in the future.

## Abbreviations

AKI: acute kidney injury
TREM-1: triggering receptor expressed by myeloid cells
HK-2 cells: human kidney-2 cells
SA-AKI: sepsis-associated acute kidney injury
LPS: lipopolysaccharide
VILI: ventilation-induced lung injury
